# Synthetic Nucleotides as Probes of DNA Polymerase Specificity

**DOI:** 10.1155/2012/530963

**Published:** 2012-06-07

**Authors:** Jason M. Walsh, Penny J. Beuning

**Affiliations:** ^1^Department of Chemistry and Chemical Biology, Northeastern University, 360 Huntington Avenue, 102 Hurtig Hall, Boston, MA 02115, USA; ^2^Center for Interdisciplinary Research on Complex Systems, Northeastern University, Boston, MA 02115, USA

## Abstract

The genetic code is continuously expanding with new nucleobases designed to suit specific research needs. These synthetic nucleotides are used to study DNA polymerase dynamics and specificity and may even inhibit DNA polymerase activity. The availability of an increasing chemical diversity of nucleotides allows questions of utilization by different DNA polymerases to be addressed. Much of the work in this area deals with the A family DNA polymerases, for example, *Escherichia coli* DNA polymerase I, which are DNA polymerases involved in replication and whose fidelity is relatively high, but more recent work includes other families of polymerases, including the Y family, whose members are known to be error prone. This paper focuses on the ability of DNA polymerases to utilize nonnatural nucleotides in DNA templates or as the incoming nucleoside triphosphates. Beyond the utility of nonnatural nucleotides as probes of DNA polymerase specificity, such entities can also provide insight into the functions of DNA polymerases when encountering DNA that is damaged by natural agents. Thus, synthetic nucleotides provide insight into how polymerases deal with nonnatural nucleotides as well as into the mutagenic potential of nonnatural nucleotides.

## 1. Introduction

Since the structure of DNA was determined [1, 2], biochemists have sought more detailed ways to study DNA and the proteins that interact with it [3, 4]. Solid phase nucleic acid synthesis of DNA molecules facilitates the site-specific incorporation of a wide range of chemically modified bases and sugar-phosphate backbones, allowing the roles of specific atoms in DNA function and recognition to be probed. Synthetic nonnatural nucleobases are useful for a variety of studies of DNA polymerase function, such as studies of DNA polymerase specificity, mutagenesis, and dynamics, as well as fluorescence resonance energy transfer (FRET) analysis of DNA polymerase interactions with DNA. The study of mutagenesis facilitated by DNA polymerases has attracted increasing interest because replication defects can lead to certain human diseases like the cancer-prone syndrome xeroderma pigmentosum variant (XPV) [5, 6] and other diseases [7–9], as well as potentially contribute to antibiotic resistance [10, 11]. Moreover, specialized damage-bypass DNA polymerases are implicated in conferring cellular tolerance to cancer chemotherapy agents that act via DNA damage, thereby decreasing their effectiveness [12–16]. This paper will focus on the ability of DNA polymerases to recognize and accept nonnatural bases either on the template strand or as the incoming triphosphate nucleotide. Much of the work discussed here will deal with A family polymerases (e.g., Klenow fragment (KF) of pol I and Taq DNA polymerase), but more recent work with Y family polymerases [17] and their ability to utilize certain nucleotide analogs will also be discussed.

**Figure 1:**
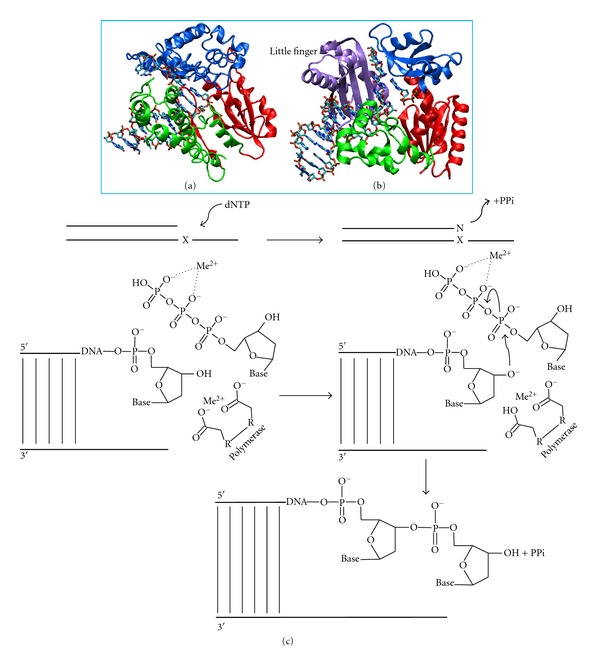
Comparison of the overall folds of (a) a replicative DNA polymerase, *Bacillus stearothermophilus* DNA pol I [30], and (b) a Y family DNA polymerase, *Sulfolobus solfataricus* Dpo4 [31]. The respective thumb domains are shown in green, palm domains are in red, and fingers domains are in blue. The little finger domain unique to the Y family DNA polymerases is in purple [31]. The “vestigial” exonuclease domain of *Bs* pol I has been omitted for clarity [32]. (c) Polymerase catalyzed DNA replication (phosphoryl transfer) reaction. Polymerization of DNA occurs at a free 3′ hydroxyl group of the deoxyribose. Polymerases use a divalent magnesium ion (Me^2+^) to coordinate the negative charges of both the phosphate groups and the aspartic acid or glutamic acid in the active site of the polymerase [26].

**Figure 2:**
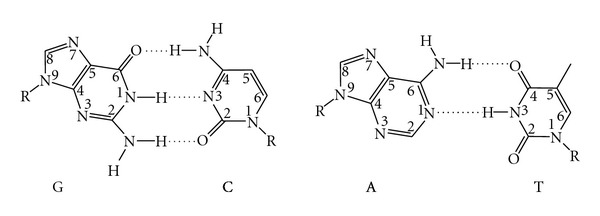
The canonical Watson-Crick base pairs. Standard numbering is indicated. Unless otherwise noted, R indicates the position of the deoxyribose in all figures.

DNA polymerases generally adopt a right-hand fold, in which the thumb and fingers bind DNA and nucleotide (Figure 1) [18, 19]. DNA polymerases add nucleotides to the growing DNA strand via nucleophilic attack of the free 3′ hydroxyl group of the DNA primer on the alpha phosphate of the incoming deoxynucleotide with release of pyrophosphate. DNA polymerase active site residues, which are usually glutamate or aspartate and are located in the palm domain, coordinate divalent magnesium ions that serve to activate the 3′-OH nucleophile (Figure 1) [20–25]. The catalytic cycle is generally accompanied by conformational changes in the fingers domain. In replicating DNA, DNA polymerases have to be able to form all four base pair combinations specifically and efficiently in order to maintain the integrity of the genome (Figure 2); however, when replication errors occur, the mismatched bases can be removed by the exonucleolytic proofreading function of DNA polymerases [26]. Replicative DNA polymerases possess an exonuclease domain that may be part of the same or a separate polypeptide that utilizes a metal-dependent mechanism to excise mismatched bases [26, 27]. The proofreading process involves translocation of the primer terminus from the polymerase active site to the exonuclease active site; after the phosphodiester bond is hydrolyzed to remove the mismatched base, the primer strand reanneals to the template so that polymerization can continue [27, 28]. Replication errors that escape proofreading can be repaired by the mismatch repair system [29].

Based on sequence conservation, DNA polymerases are divided into A, B, C, D, X, and Y families. The A and B family DNA polymerases can be involved in replication or repair, whereas members of the C family are involved in DNA replication [33]. X family DNA polymerases are involved in repair, and Y family DNA polymerases are specialized for copying damaged DNA [33] in a process known as translesion synthesis (TLS). In general, replicative DNA polymerases cannot copy damaged DNA; rather, a specialized TLS polymerase must be recruited to extend primers a sufficient distance past distortions in DNA templates to allow replicative DNA polymerases to recover synthesis [34–37]. DNA replication past damage or unusual DNA structures requires the ability to both insert a nucleotide opposite a modification in the template as well as to extend the newly generated primer beyond that position. Some polymerases may be able to insert a nucleotide opposite nonstandard bases but be unable to extend the resulting primer terminus, as discussed below. 

The four canonical bases vary in their chemical and geometric properties, but the C1′–C1′ distance of the standard Watson-Crick base pairs and the backbone C–O–P–O–C bonds remain constant regardless of the particular base pair [38]. Expansion of the nucleobase alphabet must take some of these structural considerations into account; usually nonnatural bases need to have similar geometries as the natural bases, usually but not necessarily retain some level of hydrogen bonding capabilities, and usually have *π* electron systems in order to retain the stability provided by base stacking. Hydrophobicity and base stacking interactions are also important for DNA structure [38]. 

## 2. Abasic Sites and Small Molecule Substitutions

Stable, synthetic abasic sites were first introduced into DNA in 1987 [39]. As it is estimated that 10,000 abasic sites form in each human cell per day [29], it was important to develop a stable, synthetic abasic site in order to facilitate the study of DNA polymerase interactions. Furthermore, it is informative to determine the activity of DNA polymerases in the absence of an instructional base. Takeshita et al. introduced 3-hydroxy-2-hydroxymethyl-tetrahydrofuran into DNA, which is a model for the predominant cyclic version of 2′-deoxyribose. Therefore, this analog serves as the sugar lacking the base, or an AP (apurinic/apyrimidinic) site [39]. It was shown that KF of *Escherichia coli* pol I, as well as calf-thymus DNA polymerase *α*, add dATP opposite synthetic abasic sites most frequently [39], leading to the proposal that DNA polymerases generally follow the “A-rule,” inserting A in the absence of specific coding information [40]. The cocrystal structure of KlenTaq DNA polymerase with the furan synthetic abasic site in the templating position suggests a mechanism for this, as a protein Tyr side chain fills the space left vacant by the missing base and acts as a pyrimidine base mimic [41]. Some C family DNA polymerases that are error prone and/or involved in mutagenesis can also bypass synthetic abasic sites, in the case of *Streptococcus pyogenes* by incorporating dA, dG, or to a lesser extent dC, and in the case of *Bacillus subtilis* by weakly incorporating dG and generating one-nucleotide deletions via a misalignment mechanism [42–44].* Saccharomyces cerevisiae* B-family member DNA pol zeta only weakly bypasses abasic sites [45]. Strikingly, African Swine Fever Virus (ASFV) DNA pol X is a highly error-prone DNA polymerase but is unable to copy DNA containing an abasic site [46]. Pol X cannot insert a nucleotide opposite an abasic site, nor can it extend a primer terminus containing an abasic site [46].

Because Y family DNA polymerases are known to copy noncanonical DNA structures, their proficiency at copying synthetic abasic sites has been examined in some detail. The model Y family DNA polymerase *Sulfolobus solfataricus* Dpo4 copies synthetic abasic sites mainly by incorporating dA but also by generating small deletions [47]. *E. coli* DinB (DNA pol IV) efficiently copies DNA containing a synthetic abasic site, but primarily by generating (−2) deletions [48]. *E. coli* DNA pol V (UmuD′_2_C) bypasses synthetic abasic sites by inserting primarily dA (~70%) or dG (~30%) opposite the modification [49, 50]. Even though both *E. coli* Y family DNA polymerases can copy DNA containing abasic sites, Pol V is used to bypass abasic sites *in vivo*, probably because base substitutions are generally less harmful than frameshift mutations [48]. Human DNA pol iota, which, like ASFV pol X, is highly inaccurate when replicating undamaged DNA [51], can efficiently incorporate dG opposite an abasic site but is unable to extend from primer termini containing abasic sites [52]. Human DNA pol eta copies abasic sites by incorporating predominantly dA but also dG [53, 54], whereas human pol kappa incorporates predominantly dA but also generates one nucleotide deletions [55, 56]. Y family member Rev1 from yeast incorporates dC opposite abasic sites, which have been suggested to be the cognate lesion of Rev1 [57–59]. Yeast pol alpha, replicative DNA polymerase pol epsilon, and Y family pol eta are all capable of bypassing abasic sites, whereas replicative DNA polymerase pol delta is less efficient [60, 61]. Intriguingly, yeast pol eta and KF add a pyrene nucleotide opposite the template abasic site more efficiently than adding A, likely in part because pyrene is approximately the same size as a base pair and can engage in base stacking interactions [62, 63]. Bacteriophage T4 DNA polymerase incorporates nucleotide triphosphate versions of 5-nitroindolyl, 5-cyclohexyl-indole, and 5-cyclohexenyl-indole opposite abasic sites more efficiently than it incorporates dAMP [64, 65]. Due to the complicated responses of even the relatively forgiving Y family DNA polymerases to the synthetic model abasic site, it has been demonstrated that multiple DNA polymerases may be used to bypass DNA damage efficiently while minimizing mutations [66, 67]. 

Y family DNA polymerases are able to copy DNA containing noncanonical structures ranging from abasic sites to bulky DNA adducts [68–73]. Therefore, it was of interest to determine the minimal features of DNA required for replication. Short (three or 12) chains of methylene (CH_2_) residues in the middle of canonical DNA templates were used to probe tolerance for minimal DNA backbones. *E. coli* pols I, II, and III were unable to replicate either DNA structure. On the other hand, both pols IV and V could replicate the three- or 12-methylene linker-containing DNA *in vitro*, although, in an analogous situation to abasic sites, only pol V is observed to replicate these unusual structures *in vivo* [74]. Human DNA polymerases showed more subtle differences, in that pols eta, kappa, and iota could replicate a three-methylene linker by inserting nucleotides opposite the noninstructional segment, but only pols eta and kappa could fully bypass the modified gap [75]. Pols eta and iota could insert nucleotides opposite the 12-methylene linker, whereas pol kappa had little to no activity, and none of these three polymerases could completely bypass the 12-methylene linker [75]. Clearly, at least some Y family DNA polymerases are capable of replicating non-DNA segments. 

In order to probe the size tolerance for bases in the active site, a series of dG analogs with increasingly large substituents at the *N* ^2^ position in the minor groove were constructed and used as the template base with a range of DNA polymerases. The *N* ^2^ modifications included methyl, ethyl, isobutyl, benzyl, CH_2_-napthyl, CH_2_-anthracenyl, and, in some cases, CH_2_-benzo[a]pyrenyl derivatives [76–80]. Bacteriophage T7 DNA polymerase (exonuclease^−^) and HIV-1 reverse transcriptase are both able to bypass the *N* ^2^-methyl derivative efficiently, although significantly less efficiently than unmodified DNA, but are not able to bypass any of the larger adducts [80]. Moreover, even the methyl substituent caused a high frequency of misincorporation [80]. On the other hand, each of the human Y family DNA polymerases is more tolerant of the size-expanded bases [76–79]. Rev1 is the most tolerant of *N* ^2^-dG-substitutions, followed by pol iota and pol kappa, whereas pol eta is the least tolerant, showing a decrease in activity of approximately two orders of magnitude between the CH_2_-napthyl and CH_2_-anthracenyl substituents [79]. A similar analysis of *O* ^6^-substituted bases showed that only Rev1 and pol iota could tolerate size-expanded substituents up to the benzyl substitution, but pol eta and pol kappa showed decreased activity even with an *O* ^6^-methyl substitution [79, 81]. The use of a series of well-defined synthetic base modifications provides insights into the steric constraints of DNA polymerase active sites and allows detailed comparisons to be made between replicative and damage-bypass polymerases.

## 3. Methyl-Substituted Phenyl Analogs

**Figure 3:**
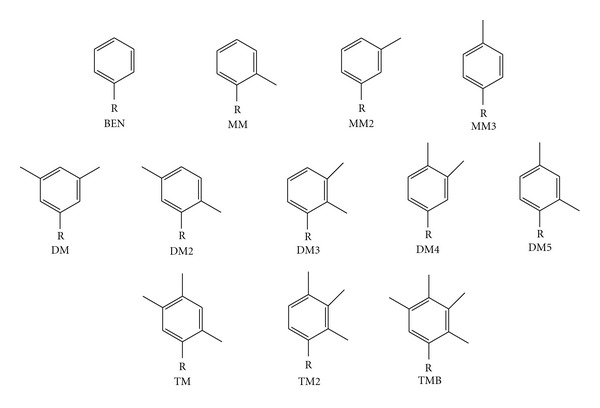
Small molecule analogs based on the benzene parent. BEN is benzene; the MM series is monomethylated at the 2, 3, and 4 positions, respectively; the DM series is dimethylated; the TM series is trimethylated; TMB is the tetramethylated benzene analog [82].

Efforts have been made to examine how DNA polymerases recognize methyl-substituted phenyl-based analogs that do not appear to be large enough to perturb DNA structure (Figure 3) [82]. There was significant self-base pairing of these substituted phenyl nucleobase analogs, which was not observed with the benzene analog [82]. Generally, in incorporation opposite these analogs, the Klenow fragment discriminates most against dCTP and dGTP, which tend to be the most hydrophilic nucleotides, while dTTP incorporation varies with the extent of methyl substitution, and dATP is added to these bases most efficiently [82]. The 2-substituted methyl-bearing phenyl groups generally favored dATP addition, but with the 3-substituted benzene rings, KF discriminated against dATP [82]. Interestingly, KF inserts dATP opposite MM1, DM2, DM5, and TMB (Figure 3) with a rate comparable to that of template dT [82]. This is hypothesized to be related not just to shape mimicry of dT in the template but to the placement of the specific substituents on the phenyl ring, which when appropriately oriented, can foster hydrophobic packing with the incoming dATP [82]. The most efficiently extended of these small substituted benzene derivatives are the ones that contain a methyl group at the 4-position [82]. Subsequent work using methoxy substituents, which unlike the methyl-substituted phenyl rings can form hydrogen bonds, suggests that positioning a hydrogen bond acceptor in the minor groove enhances both selectivity and efficiency of DNA synthesis by KF [83].

## 4. Hydrophobic Base Analogs

**Figure 4:**
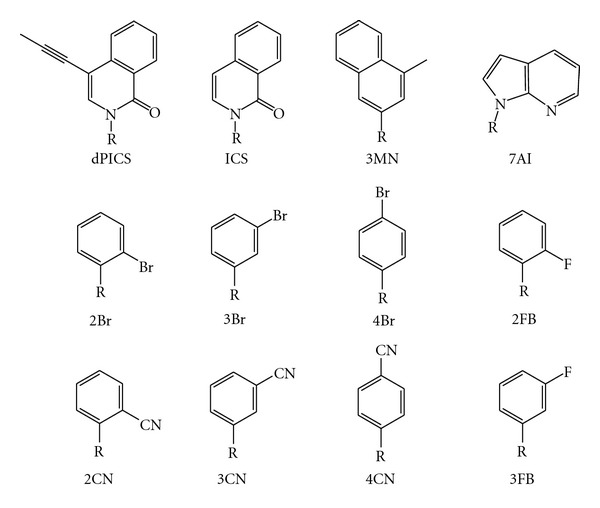
Hydrophobic nucleobase analogs: 7-propynyl isocarbostyril nucleoside (dPICS) [84]; isocarbostyril nucleoside (ICS); 3-methylnaphthalene (3MN); azaindole (7AI) [85]; bromo phenyl derivatives at positions 2, 3, and 4 (2Br, 3Br, and 4Br, resp.); cyano derivatives at positions 2, 3, and 4 (2CN, 3CN, and 4CN, resp.), fluoro derivatives at positions 2 and 3 (2FB, 3FB, resp.) [86].

The use of hydrophobic and van der Waals interactions have been the driving force behind the development of a variety of unnatural nucleobases as possible base pairing partners and to assess polymerase utilization (Figure 4) [84]. The first of these is a self-pairing base known as 7-propynyl isocarbostyril nucleoside (PICS), which stabilizes the DNA helix when paired with itself but is destabilizing when paired with dA, dC, dG, or dT [84]. The PICS base does not demonstrate structural similarity to the natural bases, but the incorporation of dPICSTP opposite PICS in the template strand by KF is more efficient than the natural bases, ranging from 20-fold more efficient than dTTP insertion opposite dPICS to ~140-fold more efficient than dGTP insertion opposite PICS [84]. KF does not extend beyond the PICS : PICS base pair, however, which is postulated to be due to a perturbation in the position of the 3′-OH of the growing primer strand caused by the nonnatural base pair [84]. 

Other hydrophobic nonnatural nucleobases are based on either the naphthalene system (Figure 4), nitrogenous base-like skeleton (Figure 4), or the skeleton of benzene (Figure 4) substituted with methyl, halide, or cyano groups [85]. While dATP is the nucleoside triphosphate most generally inserted opposite these analogs, the bromo and cyano adducts show interesting differences in KF discrimination, in that dG, dT, and dC are incorporated across from the 2-bromo derivative within threefold of the catalytic efficiency of dATP incorporation [85]. The cyano derivative at the same position leads to incorporation of dGTP ~sevenfold less efficiently than dATP, incorporation of dTTP even less efficiently, with no dCTP incorporation detected [85]. Relative to the benzene parent, only incorporation of dCTP from the 4-bromo derivative and dGTP paired opposite the 2-cyano derivative were more efficient [85]. KF primer extension after unnatural base pairing is more intriguing; specifically, 3-position substituted benzenes showed no detectable extension, with the exception of the 3-fluoro derivative which may be too small to inhibit extension due to steric hindrance (Figure 4) [85]. The base pair 4Br : 2CN was the most efficiently extended, most likely because the CN can act as a hydrogen bond acceptor and can be a driving force in primer extension [85].

## 5. Purine/Pyrimidine Mimics

**Figure 5:**
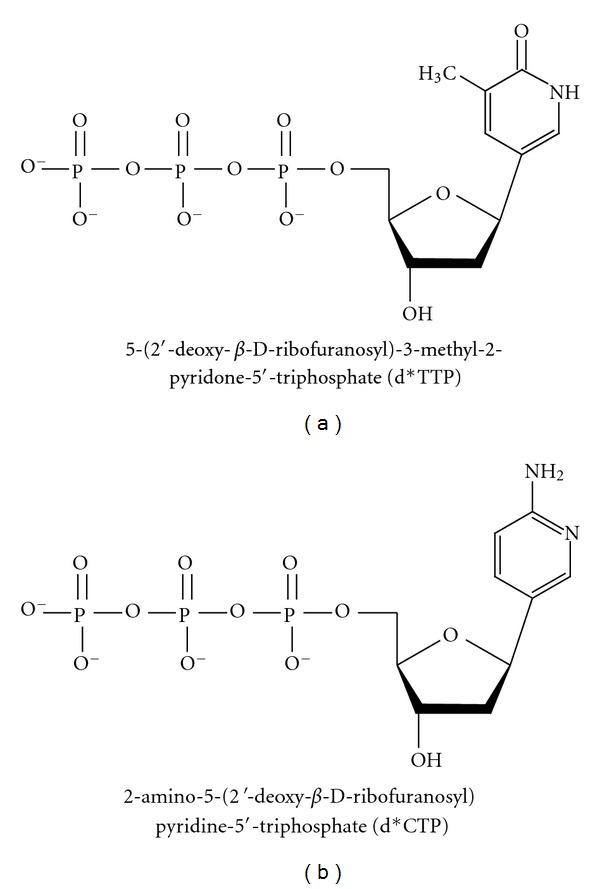
Taq polymerase activity is severely inhibited by (a) 5-(2′-Deoxy-*β*-D-ribofuranosyl)-3-methyl-2-pyridone-5′-triphosphate d*TTP and (b) 2-Amino-5-(2′-deoxy-*β*-D-ribofuranosyl)pyridine-5′-triphosphate (d*CTP), dC, and dT analogs, respectively, lacking the 2-keto groups [87].

Pyrimidine nucleotide analogs can affect polymerase activity in different ways. On one hand, pyrimidine nucleotide analogs lacking the 2-keto group can inhibit DNA polymerase activity [87]. Specifically, 2-amino-5-(2′-deoxy-*β*-D-ribofuranosyl)pyridine-5′-triphosphate (d*CTP), a cytosine analog, and 5-(2′-deoxy-*β*-D-ribofuranosyl)-3-methyl-2-pyridone-5′-triphosphate (d*TTP), a thymine analog, completely block Taq DNA polymerase from inserting them along a growing DNA strand (Figure 5) [87]. In these two analogs, in addition to the keto deletion, the C–N glycosidic bond functionality is removed and replaced with a slightly longer C–C bond, which may alter steric and electronic complementarity between the nucleotides and the polymerase [87]. These modified triphosphates, however, are tolerated by T7 RNA polymerase [88]; thus, it was concluded that the lack of the carbonyl functionality of these analogs is more responsible for the inhibition of Taq DNA polymerase than that of the longer C–C bond [87]. 

An effort to probe recognition of purines by *Bacillus stearothermophilus *DNA pol I utilized a number of aza-purine derivatives and found that substitutions of carbon at N-1 or N-3 caused the most severe defects in efficiency, whereas alterations at N-1 or *N* ^6^ resulted in loss of fidelity [89]. A similar type of analysis found that removal of the exocyclic 2-amino group of G had little effect on the efficiency of either T7 DNA polymerase or Dpo4 [90]. However, replacement of the 2-amino group by progressively larger and less electronegative substituents, F, O, and Br, led to decreasing activity by both T7 DNA pol and Dpo4 [90]. This observation led to the suggestion that the trend was due to both the size and charge of the C-2 substituent [90].

**Figure 6:**
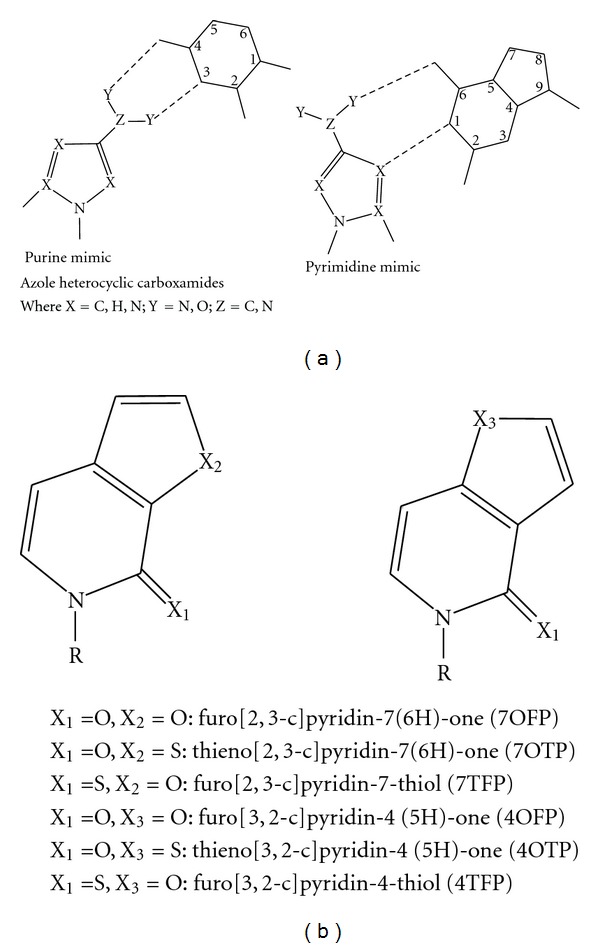
Classes of purine/pyrimidine mimics. (a) Skeleton structure of azole heterocyclic carboxamides. Depending on the position of the heteroatoms and their ability to donate or accept hydrogen bonds, these analogs can form base pairs with either a purine or a pyrimidine [91]. (b) General structure of the furo/thieno pyridinones; different heteroatoms in each position give the following compounds: furo[2,3-c]pyridin-7(6H)-one: 7OFP, thieno[2,3-c]pyridin-7(6H)-one: 7OTP, furo[2,3-c]pyridin-7-thiol: 7TFP, furo[3,2-c]pyridin-4(5H)-one: 4OFP, thieno[3,2-c]pyridin-4(5H)-one: 4OTP, furo[3,2-c]pyridin-4-thiol: 4TFP [86, 92].

Azole heterocyclic carboxamides can act as nucleobase mimics and, in fact, structurally can take on the appearance of either purines or pyrimidines (Figure 6) [91]. Because these analogs are small, they have some molecular mobility and can shift in order to adjust the hydrogen bonding patterns and electronic interactions to allow pairing with different bases [91]. Each of these azole heterocyclic carboxamides show some preference for pairing with specific incoming dNTPs, based on the position of the hydrogen bond donors and acceptors (Figure 6) [91]. For example, (1H)-1,2,3-Triazole-4-carboxamide directs the insertion of dGTP, but others do not [91]. The modified bases 1,2,4-triazole-3-carboxamide and 1,2,3-triazole-4-carboxamide, as well as 1,2-pyrazole-3-carboxamide orient in a way to promote hydrogen bonding to dC [91]. Taq DNA polymerase can utilize these analogs in PCR reactions but has different incorporation efficiencies for the different analog-dNTP pairs [91]. The presence of an azole analog in a DNA template reduces the catalytic efficiency for matched versus mismatched base pairs from 1000-fold discrepancy for natural base pairs to ~50-fold difference for base pairs involving azole analogs [91]. Therefore, these analogs are treated less stringently, but also incorporated less efficiently than natural bases by Taq DNA pol I, and demonstrate the complexity of the process of nucleotide addition, which involves electrostatic interactions, hydrogen bonding, and shape recognition [91].

 Other scaffolds for unnatural self-pairing heteroatom-containing purine mimics have been developed, known as furo or thieno pyridinones (furo[2,3-c]pyridin-7(6H)-one: 7OFP, thieno[2,3-c]pyridin-7(6H)-one: 7OTP, furo[2,3-c]pyridin-7-thiol: 7TFP, furo[3,2-c]pyridin-4(5H)-one: 4OFP, thieno[3,2-c]pyridin-4(5H)-one: 4OTP, furo[3,2-c]pyridin-4-thiol: 4TFP) (Figure 6) [86]. The goal of using these analogs is to increase the ability of the DNA polymerase to continue to extend after the analog is bypassed, which is an important step in DNA polymerization, especially for DNA damage tolerance [34–37]. The most stable base pairing of these analogs is self-pairing followed by dA, dG, dC in that order, with the sulfur moiety providing more stabilization than that of oxygen [86]. KF does not discriminate strongly when synthesizing the furo versus the thieno pyridinones as self-pairs but does exhibit differences when extending beyond the unnatural bases when they are self-paired [86]. Most of these analogs disrupted the addition of dCTP to dG at the next nucleotide position after the pyridinone self-pair, with the exception of 4TFP [86]. No natural nucleotide triphosphate is found to be inserted by KF opposite 7TFP making it the most selective. The pyridinone 4OTP is the second most selective for its self-pairing, with only dTTP a modest 1.7-fold more efficiently incorporated, and selectivity drops in the following order: 7OTP, 4TFP, 4OFP, with 7OFP being the least selective [86]. Each of these analogs, with the exception of 4OFP nucleotide triphosphate, is efficiently incorporated by KF opposite dG, with the other templating bases having lower incorporation efficiencies but that are within 20-fold of the natural DNA pairs being synthesized [86]. The extension beyond these analogs by KF polymerase increases by at least fivefold over the PICS-type analogs [86]. The purine mimic 5-nitro-indolyl-2′-deoxyribose-5′-triphosphate is known to block *E. coli* DNA replication, not by inhibiting the polymerase directly but by inhibiting the ability of the clamp loader to assemble the entire replisome by blocking ATP binding and hydrolysis [93]. However, in the Taq system, a directed evolution experiment led to the identification of a DNA polymerase variant containing multiple mutations that facilitates bypass of the 5-nitro-indole analog, while polymerization by wild-type Taq was strongly blocked [94]. The mutations were concentrated in and near the active site but were also found throughout the DNA polymerase, indicative of the multiple mechanisms by which this Taq DNA polymerase variant is able to copy unusual DNA structures [94].

**Figure 7:**
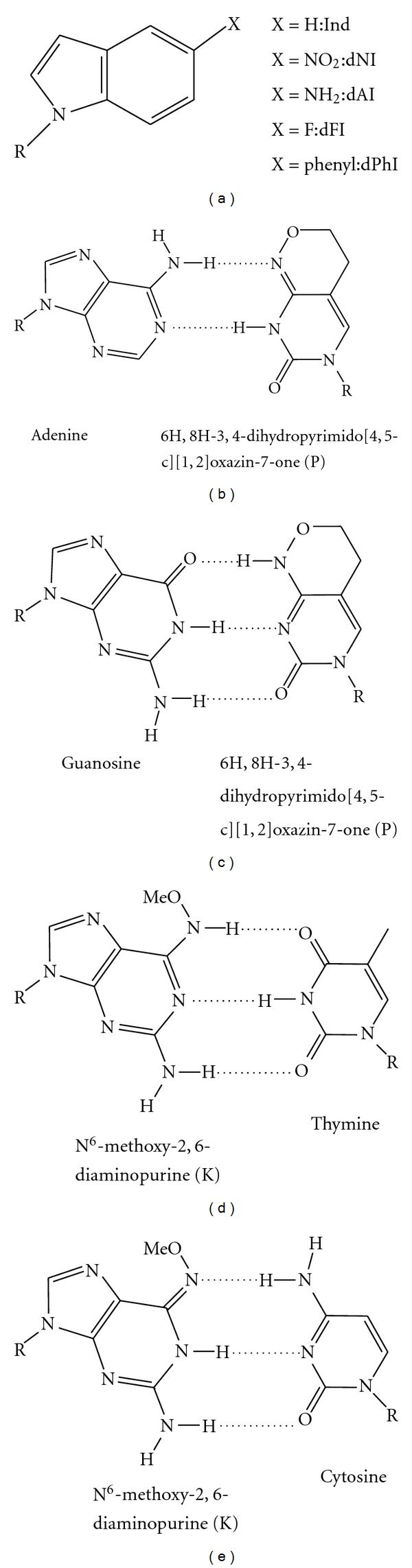
Purine and pyrimidine mimics. (a) Purine analogs based on the indole scaffold: 5-substituted indolyl-2′deoxyriboside triphosphates where X = H, indole (Ind); X = NO_2_, 5-nitro-1-indole (dNI); X = NH_2_, 5-amino-1-indole (dAI); X = F, 5-fluoro-1-indole (dFI); X = phenyl, 5-phenyl-1-indole (dPhI) [95, 96]. ((b)–(e)) Pyrimidine mimic 6H,8H-3,4-dihydropyrimido[4,5-c][1,2]oxazin-7-one (P), and purine mimic *N* ^6^-methoxy-2,6-diaminopurine (K) base pairing partners. (b) Adenine : P. (c) Guanosine : P. (d) Thymine : K. (e) Cytosine : K. The ability of these analogs to form different tautomers can result in mutations [98].

There is evidence that some unnatural dA mimics paired with abasic sites are proofread. Purines are generally added opposite abasic sites; unnatural nucleotides based on the indole scaffold substituted at the five position (Figure 7) were used to probe insertion by T4 DNA polymerase [95]. Despite the difference in size and shape, both 5-phenyl-indolyl-2′deoxyriboside triphosphate (5-PhITP) and 5-nitro-indolyl-2′-deoxyriboside triphosphate (5-NITP) are rapidly incorporated opposite an abasic site, whereas the 5-fluoro (dFITP) and 5-amino (dAITP) analogs have a very low efficiency of incorporation; the increase in *π* electrons of the former is apparently a key contributor to catalytic efficiency [95]. Two of these analogs, dNITP and dPhITP, are used as chain terminators (Figure 7) [96] but are excised more efficiently when inserted opposite a natural nucleoside as opposed to an abasic site [96]. Evidence also exists for structural changes to allow these chain terminators to be readily incorporated. Furthermore, KF proofreads bases paired with the template purine analog 4-methylbenzimidazole as efficiently as it proofreads natural mismatches; however, it is less efficient at removing 4-methylbenzimidazole from a primer terminus, suggesting that natural bases may be specifically recognized by the exonuclease active site [97].

Modified bases 6H,8H-3,4-dihydropropyrimido[4,5-c][1,2]oxazin-7-one (P) and *N* ^6^-methoxy-2,6-diaminopurine (K) are generic pyrimidine and purine mimics, respectively (Figure 7) [99]. Taq DNA polymerase copies each of these as expected: P is treated generically as a pyrimidine in the template strand, pairing with either dG or dA, and K is treated by Taq as a general templating purine, pairing with either dC or dT [99]. Taq shows a preference to use P as dT in PCR reactions, giving a dT : dC ratio of 3 : 2, while preferring to use K as dA, with an dA : dG ratio of 7 : 1 [99]. These analogs are effective as universal bases due to the prevalence of tautomeric forms, observed in nuclear magnetic resonance (NMR) experiments, that allow base pairing to multiple partners [98–100].

## 6. isoC and isoG

**Figure 8:**
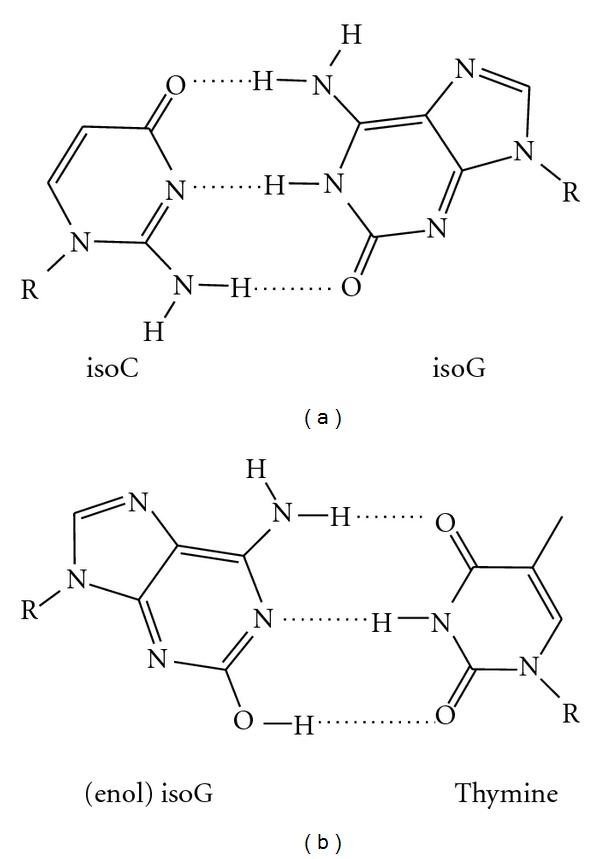
isoC, isoG, and their base pairs (a) isocytosine : isoguanosine and (b) (enol) isoG : thymine [101–106].

isoC and isoG were recognized as forming base pairs in DNA and RNA in the late eighties and early nineties (Figure 8) [101, 102] and then were accepted as a third base pair of DNA in 2003 [103]. The isoC : isoG base pair is different from its natural counterparts in the transposition of the amine and carbonyl groups on both dG and dC; however, standard Watson-Crick hydrogen-bonding is still present [104]. These analogs were first demonstrated to be useful in improving PCR efficiency [105]. isoG can take on the enol form, which base pairs with T, but it can also adopt the keto form, which base pairs readily with the thymine analog 5-methylisocytosine (MiC) [106]. The recombination protein RecA can mediate strand exchange with DNA containing iG and MiC base pairs at rates comparable to those of the natural bases, which expands the range of recombination-competent genetic material [104].

## 7. Thymidine Analogs

**Figure 9:**
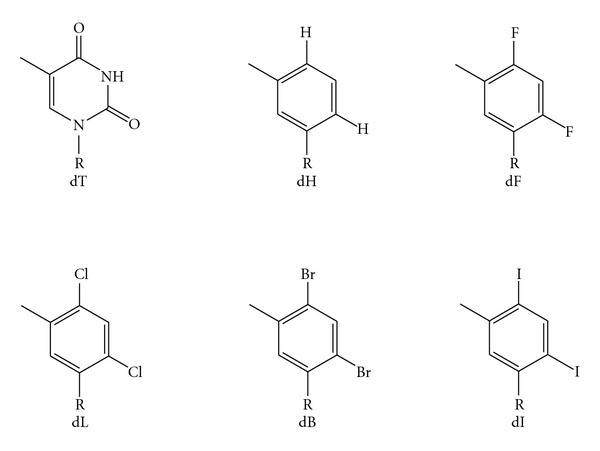
Small molecule thymidine (dT) analogs: 3-toluene-1-*β*-D-deoxyriboside (dH); 2,4-difluoro-5-toluene-1-*β*-D-deoxyriboside (dF); 2,4-dichloro-5-toluene-1-*β*-D-deoxyriboside (dL); 2,4-dibromo-5-toluene-1-*β*-D-deoxyriboside (dB); 2,4-diiodo-5-toluene-1-*β*-D-deoxyriboside (dI) [111]. The deoxyribose groups are indicated by R.

Thymidine analogs have been particularly useful for probing DNA replication. Difluorotoluene, for example, is a synthetic dT analog, in which the hydrogen bonding capabilities seen in dA : dT base pairing are reduced or eliminated (Figure 9) [107–109]. Nevertheless, this analog can serve as a very good templating base for KF [107]. Difluorotoluene promotes efficiency of insertion as the incoming nucleotide only about fourfold less than that of natural dTTP [107]. When dA at the primer end is paired with difluorotoluene as the template base, dA is removed by KF exonucleolytic proofreading as efficiently as a natural base mismatch [97]. A similar effect was observed with human mitochondrial DNA polymerase gamma [110]. On the other hand, when difluorotoluene is at the primer terminus, the relative efficiency of removal is approximately 40-fold lower than that of natural base mismatches, again suggesting that specific interactions with natural bases govern removal by the exonuclease domain [97]. Difluorotoluene is an efficient template base for KF [97]. In contrast, difluorotoluene is poorly replicated by yeast pol eta and human pol kappa [112, 113], while *S. solfataricus* Dpo4 exhibits low activity but is able to carry out primer extension on templates containing difluorotoluene [114]. 

Hydrogen bonding capacities can be retained in a structure such as 2-thioTTP, in order to improve fidelity of PCR, which can be decreased by the tautomerization of dG to form the isoG minor tautomer [105, 115]. Use of 2-thioTTP increases fidelity of those PCR reactions that include isoC and isoG [92, 105, 116] by 5% using KlenTaq DNA polymerase [115]. This is due to introducing a specific steric interaction that prevents pairing between isoG and 2-thioTTP [115]. The yellow-colored 4-Se-T is also capable of hydrogen bonding with dA and is efficiently incorporated as 4-SeTTP into DNA by KF [117].

Thymidine analogs have also been used to study the steric interactions that govern nucleotide additions. Incrementally increasing the size of the substituent in place of the carbonyl oxygen on thymidine with a series of halide substitutions (F, Cl, Br, I) demonstrates that the replicative polymerase KF has a specific “tightness” that allows for only some substitutions to be incorporated. The highest efficiency of incorporation by KF was with base pairs that are larger than natural base pairs [111]. In contrast, T7 DNA polymerase is more stringent and has an optimum that is closer to the size of natural base pairs [111, 118]. Moving the substituents around the thymidine ring and probing the activity of KF led to the conclusion that KF is remarkably sensitive to the overall shape of the template base and incoming nucleotide [119]. KF achieves maximal fidelity of incorporation with the chlorosubstituted analog 2,4-dichloro-5-toluene-1-*β*-D-deoxyriboside (Figure 9) [111]. The catalytic efficiency of KF with these analogs showed that with the increase in size by 0.66 Å (H → Cl), KF was more efficient by a factor of ~180 [111]. This trend of increasing steric hindrance with these thymidine analogs utilized by KF is consistent with the steric hindrance seen with 4′ substituted dTTP analogs noted previously [120]. In contrast, the presence of 4′ substituted T analogs in the template are well tolerated by KF [121]. The model Y family DNA polymerase, *S. solfataricus* Dbh, incorporates 4′-modified dTTP analogs relatively efficiently and binds to the analogs nearly as well as binding to unmodified dTTP [122]. Similarly, Y family DNA polymerase Dpo4 exhibits much less size selectivity than KF, as determined with halogen-substituted thymine analogs [123]. Thus, although some Y family DNA polymerases require hydrogen bonding for efficient replication, these studies confirm their generally accommodating active sites. 

## 8. Fluorescent Base Analogs

### 8.1. 2-Aminopurine

**Figure 10:**
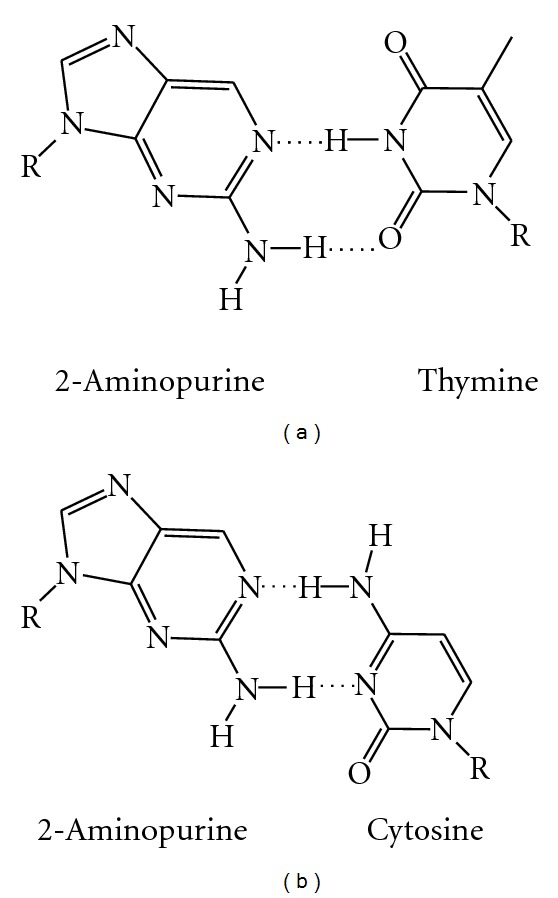
2-Aminopurine (2AP) and its base pairs (a) 2AP base paired with thymine and (b) 2AP base paired with cytosine [124].

The most common fluorescent base analog in use today is 2-aminopurine (2AP), which can form hydrogen bonds and base pair with either of the pyrimidines thymine or cytosine (Figure 10) [124]. A recent crystal structure of DNA containing a 2AP : dC base pair in the active site of the Y567A variant of RB69 DNA polymerase suggests that the 2AP : dC pair may contain a bifurcated hydrogen bond between *N* ^2^-H of 2AP and N3 and O2 of dC [125]. In this example, the Y567A active site mutation in the nascent base-pair-binding pocket is both less discriminating in the formation of mismatched base pairs and is better able to extend mismatched primer termini [125]. The modified base 2AP is commercially available and has been used to study a number of DNA-binding protein interactions including KF [126], EcoRI, DNA methyltransferase [127], endonuclease [128, 129], and uracil DNA glycosylase [130]. The fluorescence of this analog is sequence-context dependent, with the most pronounced effect occurring when the base is surrounded by other purines; much like other fluorescent nucleobases, its fluorescence is quenched when it is within DNA [131]. KF has been shown to utilize 2AP, and the fluorescence has been used to give insights into the dynamics of this protein as it synthesizes DNA [126, 132, 133]. For example, in one FRET experiment with a labeled KF, the mechanism of the fingers closing conformational change was studied [133] and was found to be influenced by the added nucleotide. Specifically, mismatched nucleotides are detected before the polymerase “closes” on the DNA suggesting that the mismatched nucleotide itself may destabilize the “open” polymerase conformation [133]. The role in the conformational change of the divalent cation (usually Mg^2+^ or Ca^2+^ but, in this case, an “exchange inert” Rh(III)) was also probed using 2AP [134], and it was found that dNTP binding in the absence of the correct ion can induce the conformational shift [134]. The ability of the ion to diffuse to the proper position before the nucleophilic attack can occur may influence the reverse conformational shift observed in the presence of the incorrect nucleotide [134].

Fluorescence spectroscopy with 2AP can be used to study DNA polymerization on a millisecond time scale, and probe single events like nucleotide addition, base pairing interactions, and subsequent excision via nuclease activity [126, 132]. Insertion kinetics have been measured for the monophosphate version of 2AP (dAPMP versus dAMP); dAPMP is found to be misincorporated at similar rates to the incorporation of the natural triphosphate dATP opposite dT by KF [126]. This makes 2AP useful in studying polymerase activity as it is misincorporated about as frequently as dA is incorporated. However, this incorporation is influenced by the sequence surrounding the primer terminus, with double the rate of misincorporation of 2AP triphosphate if the nearest neighbor to the nascent base pair is dG, dC, or dA, as compared to dT [126].

Y family polymerases also have been studied using 2AP. Dbh adds dTTP correctly opposite 2AP in the template strand and binds various DNA substrates containing 2AP with *K*_D_ values similar to those of natural DNA substrates [135]. Use of 2AP to monitor conformation changes during the base-skipping phenomenon, which can generate frameshift mutations as seen with Y family polymerases, provides evidence that the misincorporation pathway is distinct from the correct dNTP incorporation process [135]. Fluorescence from 2AP has been also used to probe the proofreading mechanism by which bases are excised via nuclease activity of phage T4 polymerase [136]. 

The analog 2AP has been used together with the base analog pyrrolo-dC as a FRET pair as the excitation and emission wavelengths of these two nucleotide probes are compatible [137], though this pair has not yet been utilized to study DNA polymerases. Pyrrolo-dC alone has been used to study DNA/RNA hybrids [138], single-stranded DNA hairpins [139], and base pair flipping [140]. Two potential drawbacks of using 2AP are the sequence dependence of its fluorescence and that it can perturb the DNA structure or be mutagenic if it forms a wobble pair with dT [124]. A 2AP : dT base pair destabilizes duplex DNA by ~8°C relative to a dA : dT base pair [141].

### 8.2. tC: 1,3-Diaza-2-oxophenothiazine

**Figure 11:**
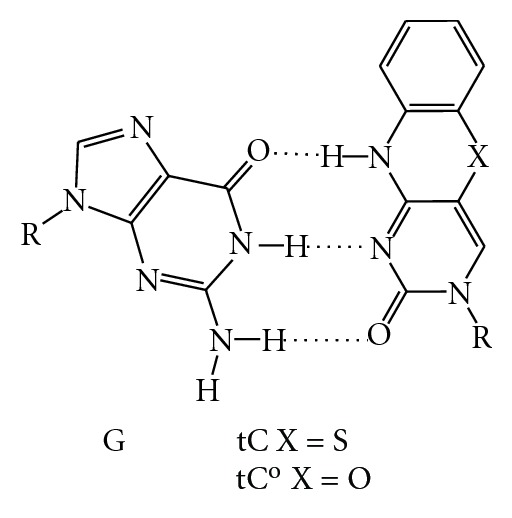
Fluorescent cytosine analogs tC and tC° form canonical base pairs with dG, do not perturb B-form DNA structure, and can pair with a FRET donor to probe DNA polymerase dynamics [144–146, 149, 150].

The synthetic cytosine analog tC was developed first by Lin et al. [142] but then used as a probe of DNA polymerases by Wilhelmsson and coworkers [143–145]. The fluorescence quantum yield of this nucleotide analog, unlike 2AP, is not sensitive to the surrounding environment [144, 146]. This base also is incorporated into DNA, shows canonical base pairing with guanosine (Figure 11), and does not perturb the B-form structure of DNA. In fact, a dG : tC base pair stabilized DNA by 3°C [124]. Different DNA polymerases have different efficiencies in utilizing tC in template DNA and in incorporating tC into the growing DNA primer strand. For example, KF utilizes template tC in preference to a template C, as KF apparently has a flexible enough active site to accommodate the extra cyclic ring system. Klenow also preferentially incorporates the tC nucleotide triphosphate in the growing DNA strand. *E. coli* DinB (pol IV), which is a Y family DNA polymerase [17], also utilizes the tC nucleotide triphosphate more efficiently than dCTP, similar to Klenow [147]. DinB also can extend from tC at the primer terminus [147]. However, DinB shows a 12-fold decrease in the catalytic efficiency of incorporation of dGTP opposite template tC as compared to the natural dC in the template strand and is unable to extend from the newly generated primer terminus [147]. Primer extension by DinB is inhibited unless the primer terminus is at least 3-4 nucleotides beyond the tC analog, which suggests that the “TLS patch” of nucleotides required beyond noncognate bases for DNA polymerases to resume efficient synthesis is shorter for a Y family DNA polymerase than for replicative polymerases. Moreover, the striking asymmetry of the DinB active site has also been observed in the case of B family DNA polymerases human polymerase alpha and herpes simplex virus I DNA polymerase when probed with nonnatural nucleotide analogs [148].

### 8.3. tC°: 1,3-Diaza-2-oxophenoxazine

The oxo-analog of tC is tC°, 1,3-diaza-2-oxophenoxazine (Figure 11) [142], which has several similar properties to that of tC in that it stabilizes B-form DNA by 3°C and it base pairs with G in a standard Watson-Crick configuration [149]. It is exceptionally bright, on average 10–50 times brighter than 2AP, 3-MI, and 6-MAP [149]. The tC° analog, like tC, can be utilized by KF and by human DNA primase [146, 150, 151]. This analog has proven useful in high-density labeling of PCR products using a deep vent DNA polymerase and therefore should be useful in biotechnology applications [151]. 

## 9. Conclusions

Nonnatural nucleotides continue to provide an important tool for the study of DNA and its interacting protein partners. In particular, DNA polymerases that are responsible for the systematic replication of DNA, whether accurate or mutagenic, are required to specifically recognize and efficiently base pair with a large number of noncanonical DNA structures. An increasingly expanding genetic alphabet of nonnatural nucleobases provides the ability to obtain an unparalleled level of detail about how DNA polymerases discriminate among many different DNA structures. From the first introduction of artificial abasic sites [39] to the use of bright nonperturbing fluorescent analogs that are used to probe polymerase opening and closing dynamics on a nascent base pair [144, 146], nonnatural nucleotides are now fully integrated into DNA polymerase research. There remains however a need for novel DNA bases that have specific properties in order to better study the interactions of DNA polymerases with DNA. In particular, efficiently generating both phosphoramidite monomers and triphosphate versions of a given modified base can be a significant synthetic challenge. The understanding of DNA polymerase specificity for synthetic nucleobases, discussed in this paper and elsewhere [28, 38, 124, 152, 153], is increasing; in the future, synthetic bases will continue to be used for a variety of purposes including probing proteins and small molecules that bind to DNA, optimizing unnatural bases for coding as a synthetic genetic code [154], synthesizing unnatural biopolymers [155], and improving the prospects of DNA as a nanomaterial and a drug target [156].
